# Concentric Array of Printed Strain Sensors for Structural Health Monitoring

**DOI:** 10.3390/s20071997

**Published:** 2020-04-02

**Authors:** Daniel Zymelka, Kazuyoshi Togashi, Takeshi Kobayashi

**Affiliations:** 1 National Institute of Advanced Industrial Science and Technology, Tsukuba, Ibaraki 305-8564, Japan; 2 NMEMS Technology Research Organization, Tokyo 101-0026, Japan

**Keywords:** strain sensor, printed electronics, structural health monitoring, sensor array

## Abstract

Civil infrastructure is expanding around the world. The ever-growing trend toward urbanization drives the demand for new investments. However, the new constructions and gradual deterioration of those already existing, especially bridges, give rise to concerns about their proper maintenance. To improve safety and drive down maintenance costs of civil structures, there is a need for inexpensive sensing systems capable of reliable and automated monitoring. In this study, we present a new concept of thin-film strain sensors arranged in an array with a concentric layout that is incorporated into a flexible substrate sheet. The designed sensor array is intended to analyze strains in the proximity of round holes made at the crack tips, found in the investigated construction elements of civil structures. In this study, the performance of the sensor array was demonstrated using measurements taken on a highway bridge in one of the largest cities in Japan. We show that it can measure local strain distribution and indicate a region with risk for crack formation. The demonstrated results show new area of potential applications for the printed strain sensors in monitoring civil structures.

## 1. Introduction

Regular maintenance inspection of civil structures such as bridges is an essential element for their proper functioning and safety. Any fatigue defects, such as cracks in susceptible regions of girders and decks, may affect their structural integrity, being a potential risk for further severe damages leading to significant ramifications. Nowadays, on-site visual inspection of bridges is still the most common method to evaluate their state and assess their level of safety. Nonetheless, the use of automated sensing systems embedded in civil structures has been growing over the last few years. The incorporation of sensors to civil structure inspection, commonly termed as structural health monitoring (SHM), relies on automated, repeated observations of their damage-sensitive elements. The main goal of SHM is to provide information about the performance, behavior, and expected lifetime evolution of the analyzed constructions [1,2]. A typical SHM system includes three main components: sensors [3], a data acquisition system [4], and software for data processing with diagnostic algorithms [5]. Currently available technologies offer several types of sensors, including fiber optics [6,7], accelerometers [8,9], and strain gauges [10,11]. The selection of sensor technology depends on the type of investigated constructions and the required analysis.

In the case of steel bridge elements, if a crack is found, the standard repair method is to drill a round hole at the crack tips. The hole is intended to stop crack propagation by eliminating stress concentration on the crack tip, thus extend the fatigue life of the bridge. The size of the crack-stop hole is usually determined according to the material properties used to build the bridge and the crack location. This hole is commonly termed as a crack-stop hole [12,13]. This reparation technique is usually a temporary solution that can serve for up to a few years. The damaged elements are usually repaired later by installing a patch plate that covers the crack and the hole. However, before reparation with the patch plate, it is not unusual for the crack to re-initiate on the other side of the hole, especially when the region with the crack-stop hole is exposed to significant load cycles. Therefore, periodic on-site visual inspections are required, which significantly increase maintenance costs. Hence, to improve the safety of civil structures and reduce maintenance costs, there is a need for reliable and inexpensive methods for automated monitoring of strains around the crack-stop holes.

In this study, we focused on local strain measurements in the proximity of damage-sensitive elements of steel bridges, where defects (e.g., cracks) were already found and temporarily repaired using the crack-stop hole. With this regard, resistive strain gauges were chosen for implementation in this work. Although strain gauges are relatively small, they can be arranged into a form of an array of sensors and provide strain measurements that cover large areas. Various concepts for the analysis of strains at large-areas have been investigated during the last years. An extensive evaluation of an array of commercial resistive strain sensors wired one by one to a flexible substrate demonstrated capabilities of such a sensing sheet to detect and quantify cracks in structural materials [14,15,16]. Another study showed a sensing sheet incorporating full Wheatstone bridge strain sensors fabricated on a flexible substrate using a photolithography etching process. The sensing sheet was analyzed during a several-hours-long field test on a concrete bridge to measure static strains [17]. Other very promising studies on the development large-area sensing systems includes capacitive sensors [18,19], and sensor arrays incorporating Lead Zirconate Titanate (PZT) piezoelectric sensors [20,21]. Printed electronics are revealed to be a suitable technique for the manufacturing of such strain sensors arrays. Using printing methods, electronic circuits adapted to complex shapes and diverse sizes of the monitored elements can be prepared easily. Printed strain sensors have been widely reported in the past few years and demonstrated for various applications, mainly in wearable devices [22,23]. However, owing to the specific features of printed electronics technology, possible application areas are much broader. The printed sensors are especially suitable for monitoring civil infrastructures, where large-area fabrication at low-cost is beneficial. Nevertheless, there is still little progress in their practical applications. In our previous work, we developed an array of screen printed strain sensors made of carbon-based ink [24,25]. The sensor array was built of 16 strain sensors arranged into a regular pattern of four rows and four columns. To compensate for the temperature sensitivity of the carbon-based strain sensors, a full Wheatstone bridge design was implemented. The evaluation of the sensor array was performed during various laboratory tests and measurements carried out on a highway bridge. The collected results demonstrate proper functioning of all sensors, even one year after installation on the bridge.

Although the currently available strain sensors are used for diverse applications and the previously reported research is promising, to date, there is no proper solution that is dedicated specially to crack-stop hole monitoring. In this study, we demonstrate a new sensing system that consists of an array of printed strain sensors arranged in a concentric layout. All sensors in the array were incorporated into one flexible substrate sheet with a specially designed shape. This makes the developed device inexpensive and easy to quickly install. The sensing system enables automated mapping of the strain levels in the proximity of the crack-stop holes. Its performance was demonstrated using measurements that were taken on a highway bridge. The resulting maps of the measured strains were used to indicate a possible direction for the crack re-initiation in the future. The demonstrated good performance of the printed sensors has great potential for practical use in monitoring civil infrastructures.

## 2. Preliminary on-site Inspection of the Crack-Stop Hole

**Figure 1:**
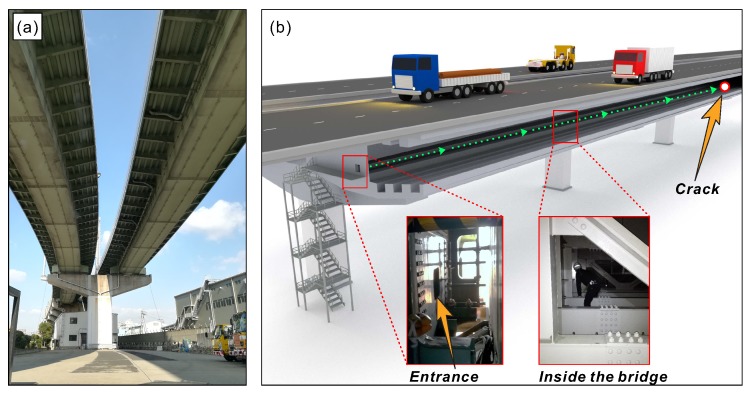
(**a**) The highway bridge with steel box girder type construction (a hollow box) analyzed in this work; and (**b**) illustration showing the crack location inside the girder.

**Figure 2:**
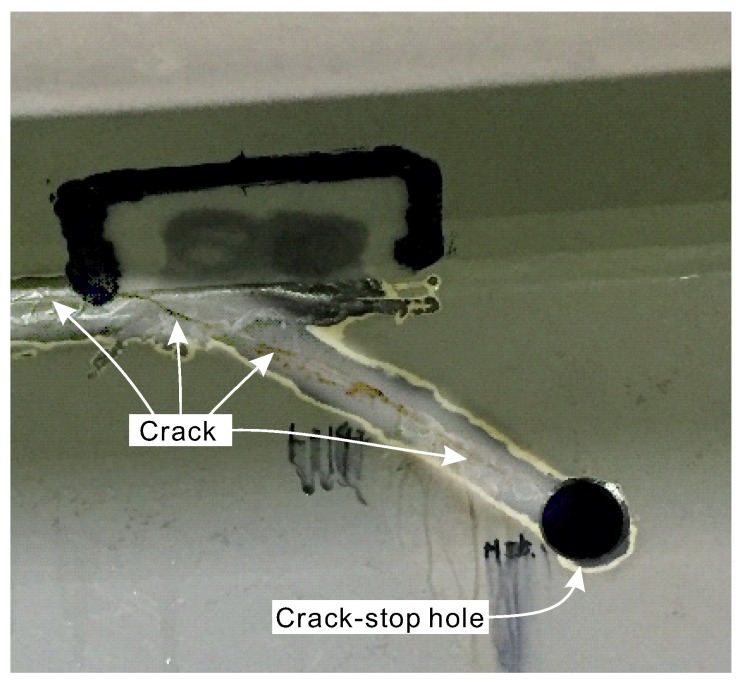
Crack and crack-stop hole made to prevent crack propagation.

**Figure 3:**
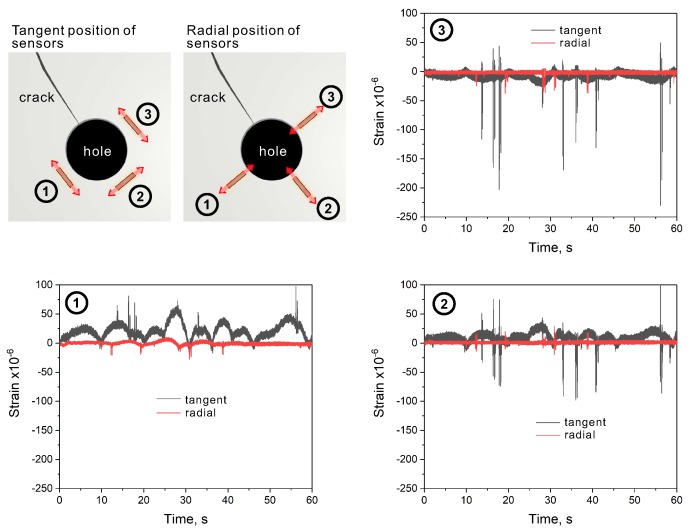
Preliminary analysis of strains around the crack-stop hole using conventional strain sensors.

The bridge that was analyzed in this study is a part of the highway infrastructure in one of the largest cities in Japan (Figure 1). Several months before we commenced this study, during a routine inspection, engineers of the bridge operator found a crack in its structure. To stop crack propagation, the bridge engineers drilled a crack-stop hole with a diameter of 2.54 cm as a standard operation (Figure 2). From that moment, the repaired spot required more attention in regards to possible crack re-initiation. We began our study from on-site inspection and preliminary strain measurements around the hole, using conventional strain sensors. The measurements were intended to assess how the orientation of sensors installed around the crack-stop hole affects the measured output signal. The sensors were arranged around the hole in three different locations, being attached in positions tangent and radial to the hole, as demonstrated in Figure 3. The analyzed bridge was constantly subjected to mechanical deformations caused by vehicular traffic. Using the sensors, we measured strains generated by the vehicles crossing over the bridge. The collected results revealed that with sensors installed radially, the measured output signal was very low. When the sensors were in the position tangent to the crack-stop hole, strains were very easily measurable. It shows that the proper orientation of strain sensors is tangent to the crack-stop hole. Based on the preliminary tests and on-site visual inspection, we determined the specifications and layout of the sensor array demonstrated in this study.

## 3. Construction of the Sensor Array

In this section, we discuss the devised concept of the sensing system used on the highway bridge. First, the resistive strain sensor in the full Wheatstone bridge configuration is briefly described. Next, an array of unit sensors arranged into a concentric layout is demonstrated together with the implemented fabrication methods. In the last part, we provide the most relevant information concerning the data acquisition system.

### 3.1. Full Wheatstone Bridge Strain Sensor

**Figure 4:**
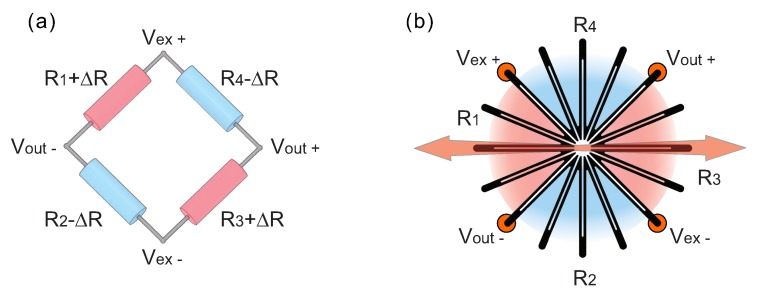
(**a**) Diagram of the full Wheatstone bridge circuit; and (**b**) full bridge strain sensor used in this work. The red arrow indicates the axis along which the sensor exhibits maximum strain sensitivity.

This study focused on the development and analysis of printed strain sensors. However, most strain sensors, especially printed, are sensitive to temperature changes. For this reason, compensation methods for temperature variations are often required. In this regard, the implementation of the full-Wheatstone-bridge circuit, demonstrated in Figure 4a is very effective in compensating for temperature sensitivity. In terms of the electrical configuration, the sensor demonstrated in this study (Figure 4b) does not differ from the conventional full-bridge sensors that are commercially available or developed by other researchers [14,17]. It is composed of four resistive sensing elements (R1, R2, R3 and R4). Between the electrodes $V_{ex}+$ and $V_{ex}-$, a constant excitation voltage is delivered (in this work 2.4 V). The relationship between the measured output voltage $V_{out}$ and the excitation voltage depends on the resistance of the four sensing elements according to the Equation (1).

(1) $$V_{out}=\left(\frac{R_{4}}{R_{4}+R_{3}}-\frac{R_{1}}{R_{1}+R_{2}}\right)V_{ex}$$

If the bridge is balanced, i.e., if all resistances are equal, the bridge generates zero output when no strain is applied. In practice, however, resistance tolerances generate some initial offset voltage. This initial offset voltage can be canceled by measuring the initial unstrained output of the circuit and its compensation in software. If all resistors are made from the same material, and thus exhibit the same thermal coefficient of resistance, under various temperatures, their electrical resistance will change equally. According to Equation (1), if the resistance change on all resistors is the same, the $V_{out}$ remains constant. This is the principle of temperature compensation by implementing the full-bridge configuration.

In terms of strain sensitivity, the senor demonstrated in Figure 4b is very similar to the commercial full-bridge sensors where two sensing elements (in this work, R1 and R3) are oriented in the direction of the intended strain sensing. When the sensor is subjected to mechanical deformations along the direction shown by the red arrow, length and cross-sectional area of sensing elements R1 and R3 also change, causing their electrical resistance change. Although the sensing elements R2 and R4 are oriented in different directions, their central axes are perpendicular to R1 and R3 and are less sensitive to the applied force (along the red arrow).

The specific design of the sensor stems from the implemented screen printing fabrication method. In the full-bridge configuration, all resistors should have at least similar resistances. However, in practice, it is challenging to achieve, especially using screen printing. In the case of printed conductive patterns, the electrical resistance may depend on the printing direction. Generally, a conductive wire directed along the printing direction has lower electrical resistance than the same line printed perpendicularly. Owing to these difficulties, we designed the sensor that incorporates 16 arms in various orientations. After the printing, the electrical resistances of the resistors (R1, R2, R3, and R4) are more uniform than in the case of a printed full-bridge sensor that has the conventional linear sensing grid oriented in two directions. We analyzed both types of screen-printed patterns, the conventional one and the one demonstrated in Figure 4b. A comparative analysis showed that in the case of printing along R2 and R4 the sensing elements R1 and R3 have higher electrical resistance. In the case of conventional pattern where the sensors were in two perpendicular directions, resistors R1 and R3 had resistance higher by about 24.9% comparing to R2 and R4. On the other hand, for the sensor design demonstrated in this work, this difference was reduced to 10.5% (R${}_{1,3}$ = 239.25 ± 1.77 kΩ, R${}_{2,4}$ = 216.45 ± 7.85 kΩ). Although screen printing of full-bridge strain sensors with equal resistances of sensing elements remains challenging, in Section 4.2, we show that, despite the differences in the resistances, the developed sensor exhibits effective compensation for temperature changes. The characterization of the printed full-bridge sensor has been described in our previous work [24,26]. Nonetheless, additional detailed analysis is still required and currently ongoing. Although we used our original sensor design in this study, the conventional full-bridge sensor can be used in the same way, especially if other fabrication methods are used.

### 3.2. Concentric Sensor Array

**Figure 5:**
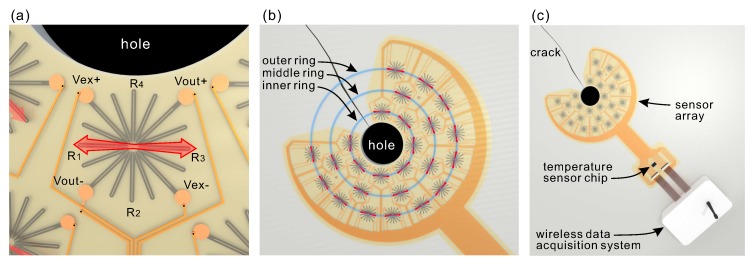
(**a**) Design of unit sensors that are incorporated into the sensor array. $V_{ex}$ and $V_{out}$ correspond to the electrodes where the excitation voltage and output voltage terminals are connected, respectively. R${}_{1–4}$ show the particular resistive elements of the printed structure that complete the full-Wheatstone-bridge circuit. The red arrow demonstrates an axis along which the sensor exhibits maximum strain sensitivity. (**b**) The layout of the concentric sensor array. The sensors were arranged into three rings. The substrate design allows proper alignment of the sensors around the hole. (**c**) Concept of the measuring system incorporating the concentric strain sensor array and wireless data acquisition system.

As demonstrated in Section 2, a key point for the proper functioning of the sensing system is an appropriate arrangement of the unit sensors within the array. Our preliminary measurements showed that the proper orientation of sensors is tangent to the hole. Moreover, the propagation direction of fatigue cracks emanating from the hole is expected to be straight along the radial direction for a specimen with a hole, subjected to mechanical deformation [13,27]. In this study, we used resistive strain sensors in a full Wheatstone bridge configuration (Figure 5a). The full-bridge sensor exhibits maximum strain sensitivity in one specific direction, shown by the red arrow. Thus, the arrangement of the sensors tangent to the hole, stems from their optimal orientation along which the sensors exhibit maximum sensitivity.

Although we can predict that the propagation of a crack will be straight along the radial direction [13,27], it is difficult to predict the point around the hole from which the crack may initiate. In real-life conditions, construction elements of bridges are subjected to mixed deformation modes [28], at angles that depend on the specific construction of the bridge. The accurate determination of load direction, and thus the prediction of susceptible regions around the hole is difficult without numerical simulations. However, such simulations would be required individually for each specific case. Due to the complex construction of some bridge elements, it would be challenging and very costly. To address this problem, many sensors, covering a large area around the hole, were used.

Hence, while designing the large 25 sensor array, we arranged them so that, after the installation, all of them were oriented tangent to the crack-stop hole. Moreover, besides the tangent positions, the sensors were arranged into three rings that were concentric with the center of the crack-stop hole (Figure 5b). The sensors on the inner ring of the array are the most important to indicate the susceptible region with the risk of crack formation. The sensors on the middle and outer rings are supplementary and intended to monitor crack propagation, in case it appears.

The layout of the array and the shape of the substrate were specially designed to allow proper alignment of the sensors around the hole and to avoid a direct installation of sensors on the crack (Figure 5b). This is because cracks found on steel elements of bridges are usually exposed to significant strain levels. Direct installation of the sensor array on the crack would most likely cause severe damage to the sensors. The region surrounding the hole was the subject of our analysis. Having devised the concept for the sensor array, we began its fabrication.

### 3.3. Fabrication Process

**Figure 6:**
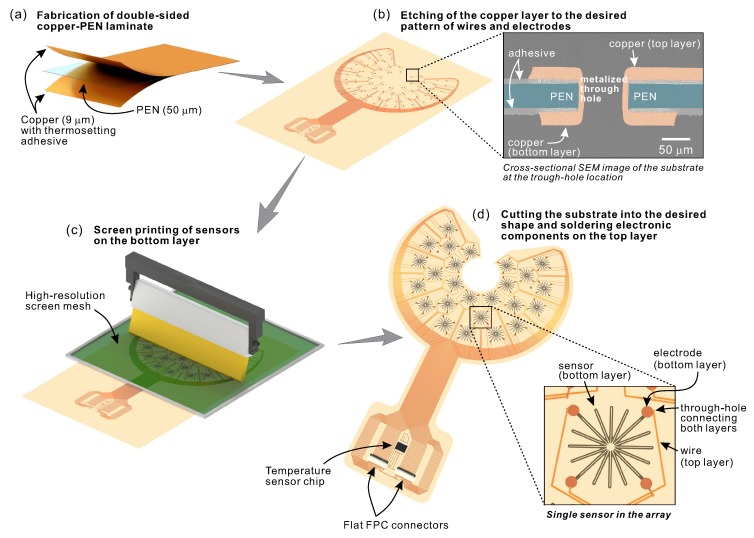
(**a**) Dry lamination process of PEN substrate between two 9-μm-thick copper sheets; (**b**) wet etching of the laminate to form the desired pattern of copper wires and electrodes; (**c**) screen printing of an array of 25 strain sensors; and (**d**) cutting the substrate into the desired shape and soldering the electronic components.

**Figure 7:**
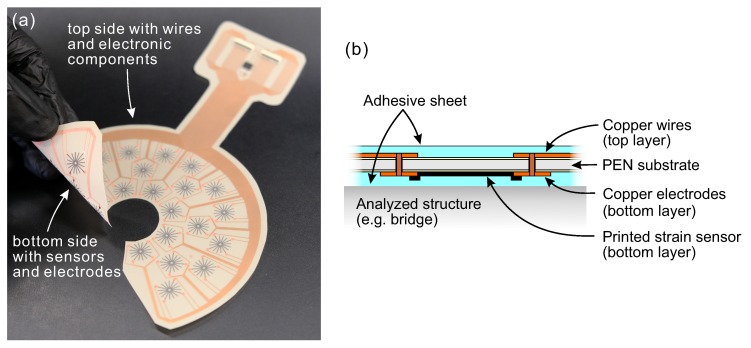
(**a**) Photograph of the sensor array; and (**b**) side view of the sensor structure.

The fabrication process of the sensor array was divided into four main steps that are shown schematically in Figure 6. The laminate of 50-μm-thick polyethylene naphthalate (PEN) sheet between two 9-μm-thick copper sheets was prepared in a dry lamination process. To form the desired pattern of copper wires and electrodes, the laminate was subjected to wet etching in ferric chloride. The minimum width of the created lines and pitch between them were 0.2 and 0.15 mm, respectively. The connections between the copper layers were made by making through-holes and electroplating them. Next, an array of 25 strain sensors was screen-printed using a conductive carbon ink (Toyobo DY-200L-2) on the bottom side of the substrate. The carbon ink was selected based on our previous studies [24,26]. Carbon-based sensors provide desirable sensor properties yet are very cost-effective. A stainless steel mesh (Asada Mesh HS-D 650/14) was used for high-resolution printing. The sensors were cured in a conventional oven at 130 °C for 30 min. After the curing, the measured thickness of the printed sensors was 4.43 ± 0.24 μm. In the last step, the substrate was cut into the desired shape, and the connectors (Molex 502598-3393) and a temperature sensor chip (Analog Devices TMP36) were soldered to their proper locations in the electronic circuit. While the strain sensors were printed on the bottom side of the substrate, the electronic components were attached to the top side. Such construction was intended to direct the sensors face-down towards the monitored structure so that their functioning was not affected by the mechanical properties of the substrate. The fabricated device and side view illustration are demonstrated in Figure 7. The diameter of the unit sensors was 10 mm. The maximal width and length of the entire device were 118 and 176 mm, respectively. These dimensions were selected according to the specific testing conditions on the highway bridge, i.e., size of the crack-stop hole (2.54 cm), and available space for sensor installation. However, the versatility of the printed electronics process makes it relatively easy to prepare a diverse array of sensors for various applications. During the measurements, the sensor array was bonded to the monitored structures using a 200-μm-thick epoxy-based adhesive sheet. The same type of adhesive sheet was used as a protection layer to cover the sensor array on the top side, after it was attached to the bridge.

### 3.4. Data Acquisition System

**Figure 8:**
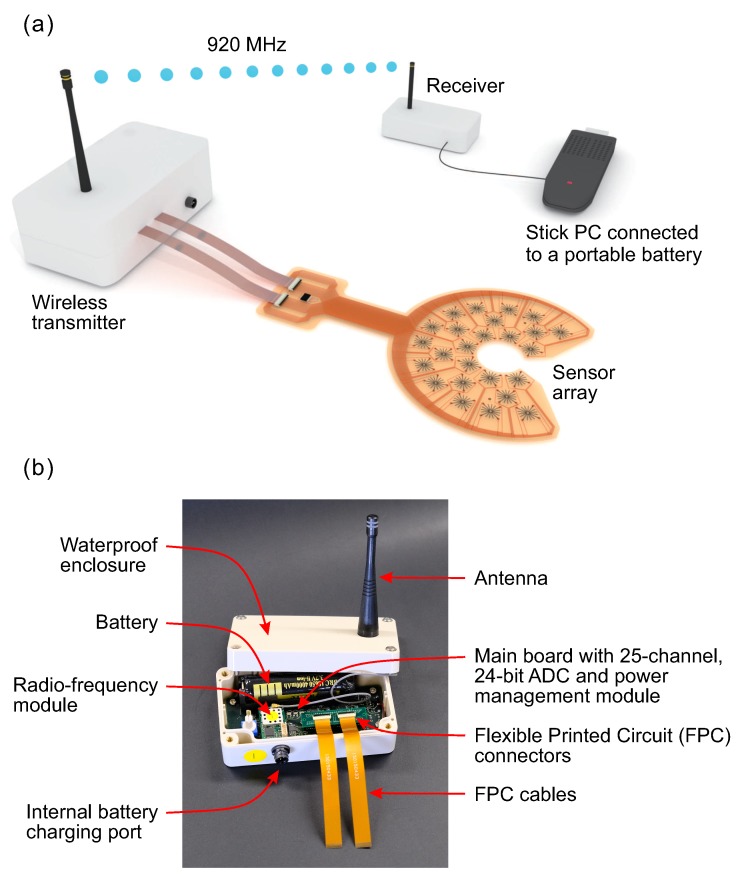
(**a**) Measurement setup composed of a wireless transmitter, sensor array, receiver, and low power Stick PC; and (**b**) photograph of the wireless transmitter incorporating 25-channel, 24-bit ADC.

The data acquisition system (DAQ) was custom made for this work by Global Interface Technologies, Inc. It was composed of a wireless transmitter (920 MHz) and receiver connected to a low power computer (Intel Compute Stick PC) (Figure 8a). The transmitter was equipped with a 25-channel, 24-bit analog-to-digital converter (ADC) and was connected to the sensor array via the Flexible Printed Circuit (FPC) cables and connectors. During the measurements, the differential output voltage from all 25 strain sensors was recorded simultaneously and stored in the internal memory of the Stick PC. The entire data acquisition process was controlled using specially prepared software (LabView, National Instruments). When taking measurements on the bridge, data were automatically collected at a scheduled time. The transmitter had a built-in battery and power management module (Figure 8b). To save energy, the transmitter could be turned to sleep mode, and automatically activated at a scheduled time, during the period of performing measurements. If needed, the battery can be charged via the built-in charging port, using a solar panel or an AC adapter for long-term operation. Although we used the custom made DAQ in this study, any other commercial measuring systems capable of 24-bit differential voltage measurements can be used equally.

## 4. Laboratory Tests—Evaluation of the Full-Bridge Sensor

### 4.1. Strain Sensitivity

**Figure 9:**
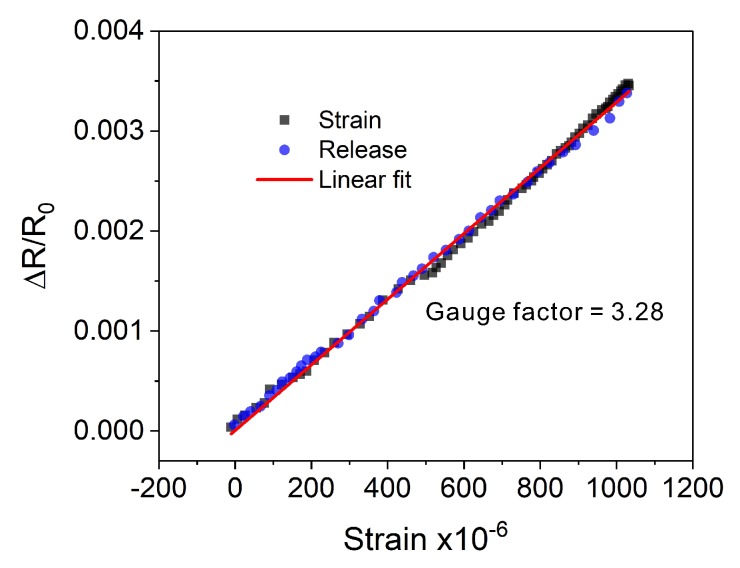
Strain sensitivity and linearity test of the printed strain sensor.

To evaluate strain sensitivity, our sensors were calibrated based on a reference strain measurement that was taken using a conventional strain sensor (Kyowa KFG-10-120-C1-11L1M2R). Both sensors were bonded to a metal plate that was subjected to bending deformations. The calibration process was carried out using a tensile test machine (Aikoh FTN1-13A). The output signals were measured simultaneously and compared. The collected results are demonstrated in Figure 9. Based on the recorded data, the sensitivity (gauge factor (GF)) of the printed sensors was calculated to be 3.28, which is slightly higher than that of the conventional strain gauges (GF ≈ 2.1), including the commercial full-bridge sensors. In the case of printed sensors, the sensitivity can be changed by the use of composite materials or the implementation of a sensor with a modified microstructure [29]. Nonetheless, the achieved sensitivity of 3.28 is sufficient for practical applications within the framework of SHM. Moreover, the sensor exhibits good linearity and no hysteresis within the analyzed strain range.

### 4.2. Temperature Sensitivity

**Figure 10:**
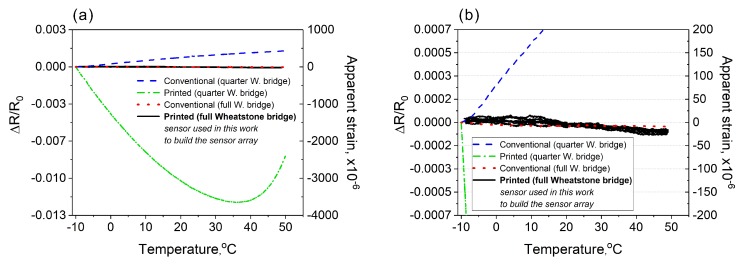
(**a**) Comparative analysis of temperature sensitivity between the printed strain sensors and conventional strain gauges. Sensors were analyzed in two different electrical configurations (quarter- and full-Wheatstone-bridge). (**b**) Detailed plot demonstrating the effectiveness of the full-bridge sensors. In the case of the printed full-bridge sensor, the plot demonstrates three cycles within the investigated temperature range.

Besides the strain sensitivity, the sensors were evaluated in terms of their sensitivity to temperature variations. Figure 10 demonstrates a comparative analysis of the temperature sensitivity carried out using printed sensors made of the carbon ink and commercially available strain sensors made of a copper-nickel alloy (Kyowa KFG-10-120-C1-11L1M2R and Omega SGT-4/1000-FB11, full-bridge sensor). The sensors were analyzed in two different electrical configurations: the quarter-Wheatstone-bridge (no compensation for the temperature variations) and the full-Wheatstone-bridge (with compensation). The measurements were conducted in an environmental test chamber (Espec SH-221) in a temperature range from −10 to 50 °C. To show how temperature change affects the measured strain values, on the Y-axis beside the resistance change, we calculated the apparent strain (using specific gauge factors for each sensor). During the test, the sensors were not subjected to any mechanical deformations. Thus, the recorded strain changes were mainly due to the temperature change. The difference between the quarter bridge and full bridge connections is clearly noticeable. Especially in the case of the printed sensor where with the quarter-bridge, the apparent strains were in the range of thousands of μϵ. Nonetheless, the collected results demonstrate that, even for the printed sensor, the full-bridge configuration enables sufficient temperature compensation (Figure 10b). It makes the developed sensors suitable for practical applications to dynamic strain analysis within the framework of SHM.

## 5. Practical Evaluation of the Sensor Array on the Highway Bridge

**Figure 11:**
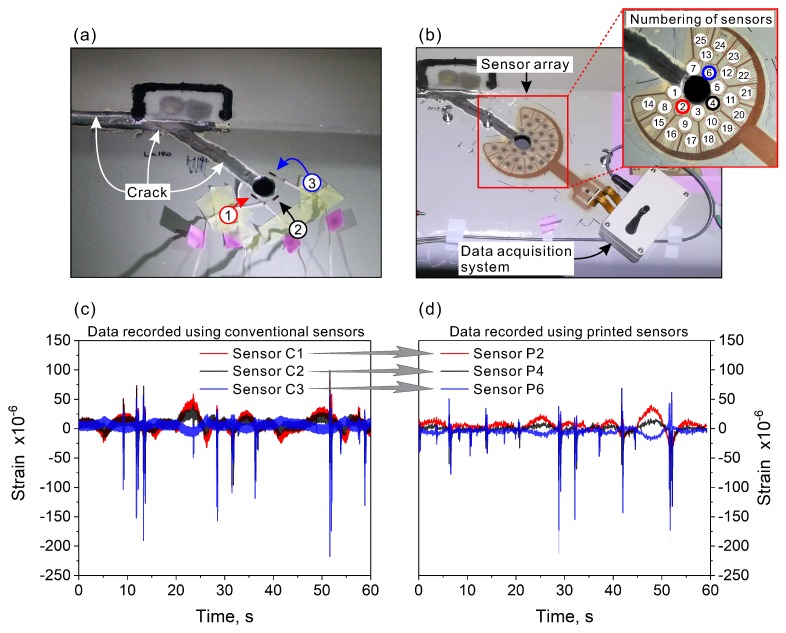
(**a**) Conventional foil strain gauges attached tangent to the hole, used for the preliminary strain measurements around the hole; (**b**) the developed array of 25 printed strain sensors and wireless data acquisition system used for strain analysis in the proximity of the crack-stop hole; (**c**) results collected using conventional strain sensors; and (**d**) results obtained using the printed strain sensor array.

In the first step, the printed sensor array was evaluated based on the reference strain measurement taken with three conventional strain sensors arranged tangent to the hole, as demonstrated in Figure 11a. For easier distinction between the printed and conventional sensors, in this paper, “C” refers to conventional sensors, while “P” the printed sensors. The printed sensor array and data acquisition system were bonded to the bridge structure (Figure 11b). Although the array has 25 unit sensors, to compare its functioning with the conventional sensors, only three printed sensors from the array (P2, P4, and P6) were analyzed. The location of these sensors was the same as that of the conventional sensors C1, C2, and C3. With both types of sensors, 60-s-long data samples were recorded. Figure 11c shows strain variations recorded using the conventional sensors. Comparing these results to those registered with the printed sensors (Figure 11d) shows that the collected data are very similar. The strain variations for both types of sensors at the corresponding locations (C1–P2, C2–P4, and C3–P6) were consistent in terms of amplitudes and measured strain patterns. The above results show that the developed concentric sensor array works as it was intended.

**Figure 12:**
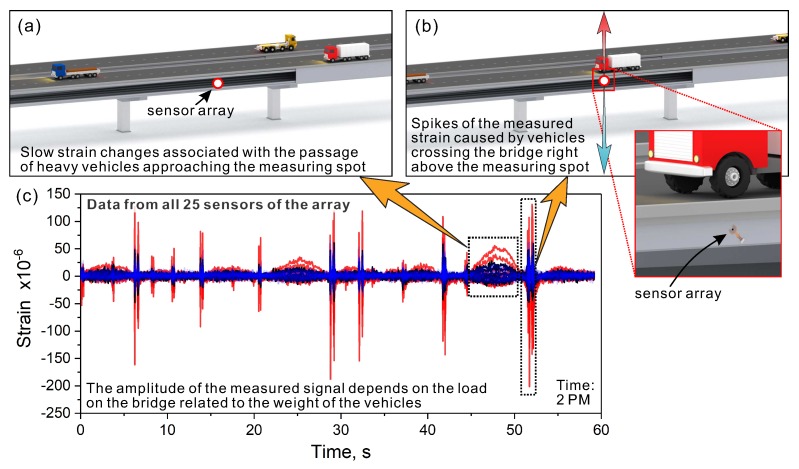
Schematic illustration of two types of strain changes registered on the bridge: (**a**) slow strain changes associated with the passage of heavy vehicles approaching the measuring spot; (**b**) spikes of strain recorded when the vehicles were right above the measuring spot; and (**c**) 60-s-long dataset of a strain measurement carried out using all 25 printed sensors in the concentric array. Data were recorded at 2 PM.

In both cases, for the conventional and printed sensors, two types of strain changes were observed. There were slow changes and sudden spikes with relatively large strain amplitudes. A brief explanation on this phenomenon can be given based on our on-site observations and measurements. The slow strain changes were associated with the passage of heavy vehicles approaching the measuring spot (Figure 12a). On the other hand, the spikes of the measured strain were observed when the vehicles crossed the bridge right above the measuring spot (Figure 12b). The spikes had much larger strain amplitude in comparison to the slow strain changes and were associated with the dynamic response of the bridge to vehicular traffic. The complete dataset recorded using the 25 printed sensors is shown in Figure 12c.

**Figure 13:**
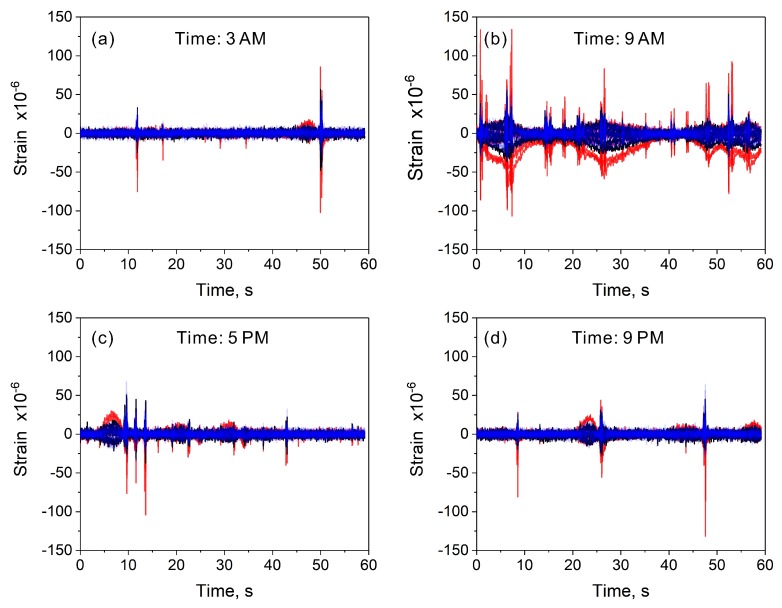
Selected data demonstrating daily strain changes on the analyzed bridge structure.

To verify how the volume of daily traffic affects the measured strains, we analyzed the data collected at different periods of the day. In this study, 60-s-long measurements were performed automatically every 1 h, for five days, by a programmable data acquisition system. To provide a general overview of the daily strain changes, we compared the results recorded at 2 PM (Figure 12c), with those taken at 3 AM, 9 AM, 6 PM, and 9 PM (Figure 13). It can be seen that at 3 AM (Figure 13a) the traffic volume was significantly reduced. Because of very early hours, such a result was expected. Based on the strain spikes, we could distinguish between single vehicles crossing the bridge. Slow strain changes were not registered during this time. The strain variations become significant during morning hours, especially around 9 AM (Figure 13b). Numerous spikes with long and slow strain transitions were observed. This bridge response is consistent with typical traffic conditions in large urban agglomerations, where the traffic intensifies during morning hours. In the afternoon, the traffic volume decreases but remains high (Figure 12c). From the evening hours, we observed a gradual decrease in the traffic volume (Figure 13c,d). The above results demonstrate the versatility of the developed sensing system. Besides the strain measurements, it can be used for the quantitative analysis of traffic conditions.

**Figure 14:**
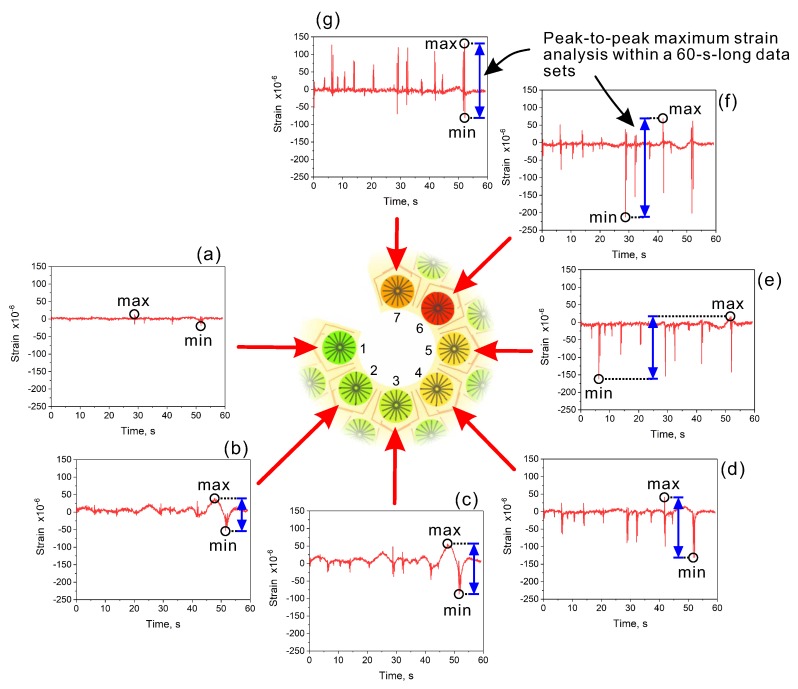
Demonstration of the proceeding scheme for the data analysis recorded by the unit sensors of the concentric array. To simplify the illustration, we showed a signal from the first seven sensors instead of all 25. During measurement, the analysis was done automatically by the developed software.

However, in this work, the analysis of the maximum strains measured around the crack stop-hole was the particular point of interest. The analysis was intended to provide valuable information on strain evolution, thus indicate possible regions for the crack re-initiation in the repaired bridge elements. As described above, the measurements were carried out automatically every hour. This period can be freely changed, and for most practical applications can be performed less frequently. However, in this study, the measurements were scheduled for only five days. To increase the amount of data, the measuring system was set to operate every 1 h. After each measurement, the computer program analyzed the recorded 60-s-long dataset, determining the maximum and minimum strain values. The analysis was done individually for all of 25 sensors of the array. Once the maximum strain levels were found for each sensor, a map of the maximum strain values was created using color indicators corresponding to the measured strain values. This process is schematically demonstrated in Figure 14. Although the analysis was performed for all the 25 sensors, in this example, the demonstrated data is from sensors P1–P7. Those sensors were closest to the hole, thus large strains were measured.

Starting from P1 (Figure 14a), negligible strain variations were observed. This region of the crack-stop hole was subjected to the lowest strain variations. In sensors P2 and P3 (Figure 14b,c), visible strain changes mainly associated with slow strain changes were observed. From P4 to P7 (Figure 14d–g), the spikes of the measured strain associated with the dynamic response of the bridge to vehicular traffic become dominant. A maximum strain range of about 300 $\times \;10^{-6}$ was detected on P6 (Figure 14f).

**Figure 15:**
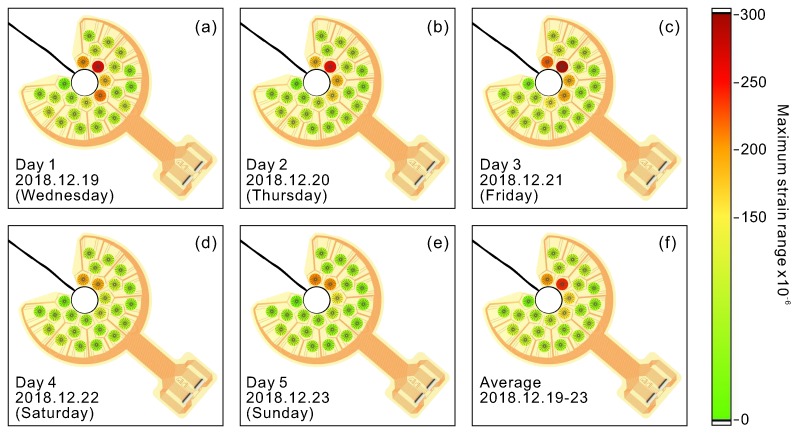
Daily analysis of maximum strain distribution around the crack-stop hole.

This data analysis approach was used for all recorded measurements during the five test days. In Figure 15, a summary of all the results taken day-by-day is presented in the form of maps representing the maximum strain ranges registered on each day. While during the weekdays the maximum strain levels remain similar (Figure 15a–c), the maximum measured strains had a noticeably lower value of about 200 $\times \;10^{-6}$ (Figure 15d,e) during the weekend. The reduced strain values were most likely associated with the reduced traffic volume of heavy vehicles during the weekend. Moreover, the trend with registered higher strain levels on the upper-right side of the hole (associated with the dynamic response of the bridge) and the slow strain changes on the lower-left side was observed in all recorded measurements. The reason the slow strain changes were observed on the lower-left side of the hole and the spikes on the opposite side were most likely as a result of the specific construction of the bridge, materials used, and the crack position in the proximity of the measuring spot. Nonetheless, thanks to the developed sensing system, we were able to localize a region with enhanced strain levels that was within the proximity of sensor P6. According to the analysis, this region faces the risk of crack re-initiation in the future. Besides the strain analysis, our sensing system can measure temperature on the analyzed spot. The results demonstrating the recorded daily temperature variations are shown in Figure S1 (see the supplementary materials). Overall, the developed device demonstrates broad capabilities that can be useful in diverse applications. The collected results show the suitability of the thin-film printed strain sensors for practical applications in the monitoring of civil infrastructures.

## 6. Conclusions

In this study, a sensing system composed of 25 printed strain sensors arranged in an array with a concentric layout was demonstrated. The sensors were incorporated onto one flexible sheet of a substrate. The full Wheatstone bridge configuration of unit sensors enables effective compensation for temperature variations, and the concentric design of the sensor array allows proper analysis of strains in the proximity of round hollow objects (including crack-stop holes) in various civil structures. The sensor array was fabricated using inexpensive carbon-based materials. It can be easily redesigned to various shapes that may be required for other types of civil structures. Demonstrated application possibilities include local mapping of strain distribution and analysis of daily traffic volume on the monitored bridges. Using our concentric sensor array, we were able to distinguish between different types of deformations subjected to the analyzed area, i.e., the slow strain changes associated with the passage of heavy vehicles and the strain spikes related to the dynamic response of the bridge to vehicular traffic. The collected strain distribution data were used to localize the region with a potential risk for the occurrence of fatigue defects in the future. The reported results show a new practical application area for the printed strain sensors in monitoring civil structures. The versatility of the printed sensors, with their demonstrated proper performance, indicate great potential for broader application.

## Supplementary Materials

The following are available online at https://www.mdpi.com/1424-8220/20/7/1997/s1, Figure S1: Daily temperature changes recorded inside the box girder of the bridge (2018.12.19–23).
