# Supplementary material for: Concentric Array of Printed Strain Sensors for Structural Health Monitoring
Source: Sensors (Basel). 2020 Apr 2;20(7):1997. doi: 10.3390/s20071997 (PMC7180977; doi:10.3390/s20071997)
Supplement: Supplementary file 1 [file sensors-20-01997-s001.pdf]

Supplementary Materials: Concentric Array of Printed Strain Sensors for Structural Health Monitoring

Daniel Zymelka [1]*, Kazuyoshi Togashi [2] and Takeshi Kobayashi [1]

1. Temperature changes during the five days long measurements on the bridge

Temperature measurements were carried out using the temperature sensor chip that was integrated on the top layer of the flexible substrate. The measurements were performed simultaneously with the strain analysis using the same wireless data acquisition system, that had a dedicated input for the temperature sensor. The collected data shows typical daily temperature variations (Fig. S1).

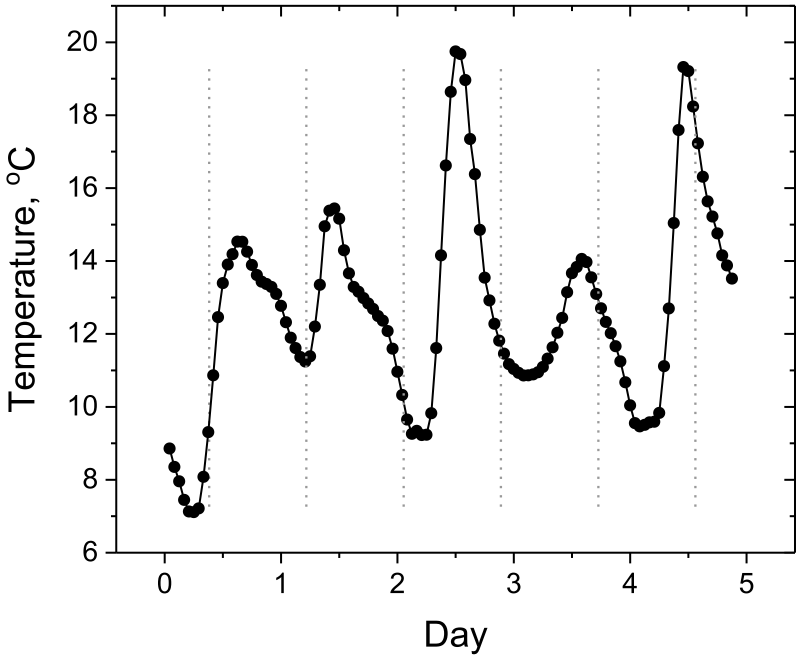

Figure S1. Daily temperature changes recorded inside the box girder of the bridge. (2018.12.19-23)
